# Social Isolation and Suicidal Ideation of Older People: The Buffering Effects of Neighborhood Social Cohesion

**DOI:** 10.1093/geroni/igab046.947

**Published:** 2021-12-17

**Authors:** Choi Bomi, Hey Jung Jun, Susanna Joo

**Affiliations:** 1 Yonsei University, Seoul, Seoul-t'ukpyolsi, Republic of Korea; 2 Yonsei University, Seodaemun-gu, Seoul-t'ukpyolsi, Republic of Korea

## Abstract

This study aimed to examine the buffering effect of neighborhood social cohesion on the association of social isolation and suicidal ideation among Korean older people. The sample was older adults who were 65 years old or older and participated in the Korea Health Survey 2017 collected by the Center for Disease and Prevention (N=67,835). Social isolation was measured with three indicators: living-alone, contact isolation (less than weekly contact with family, friends, or neighbors), and participation isolation (less than monthly social organization attendance). Neighborhood social cohesion was measured with two indicators: trust in neighbors and the welfare budget ratio to represent social capital and social inclusion capabilities, respectively. Multilevel logistic regression analyses were performed to estimate the dynamic relationships between social isolation, neighborhood social cohesion, and suicidal ideation. Results of the main effect indicated that social isolation is a significant risk factor for suicidal ideation and neighborhood social cohesion works as a protective factor against suicidal ideation. Results of cross-level interactions showed that the welfare budget ratio moderated the association between participation isolation and suicidal ideation (OR=0.960, p<.001). The negative effect of participation isolation was reduced as the welfare budget ratio of the neighborhood increased. Neighborhood social cohesion indicators did not moderate the association between the remaining types of social isolation and suicidal ideation. The evidence from this study highlights the importance of social welfare expenditures when building suicide prevention interventions and age-friendly communities.

